# Physiological Alterations in Local Iraqi Sheep Affected by Ruminal Impaction: Insights from Biochemical, Electrolyte, and Oxidative Markers

**DOI:** 10.12688/f1000research.174402.1

**Published:** 2026-01-23

**Authors:** Sabea Khamees Abed, Mustafa A. Saud, Ahmed Emad Abood, Maysam Abdulrahman Ghazi

**Affiliations:** 1 College of Veterinary Medicine, University of Fallujah, Al-Fallujah, Al Anbar Governorate, Iraq

**Keywords:** Keywords: ruminal impaction; sheep; metabolic acidosis; oxidative stress

## Abstract

**Background:**

Ruminal impaction is a serious digestive disorder in sheep, leading to systemic metabolic acidosis and, as a consequence, widespread physiological disturbances. Thus, the objective of this study was to estimate biochemical, electrolyte and oxidative parameters in a local Iraqi sheep that was suffering from ruminal impaction.

**Methods:**

A case control study was performed in 20 adult female sheep (Awassi sheep breeder) (10 ruminal impaction and 10 control) diagnosed with in Fallujah, Iraq, based on clinical signs and confirmed by laboratory findings of hyperkalaemia. The control group consisted of sheep of age, sex and weight consistent with clinically normal sheep. At the end of the experiment, blood samples were collected, and serum was analyzed for electrolytes, biochemical and oxidative markers using established laboratory methods.

**Results:**

The impacted animals showed significant increases (P≤0.05) in alanine aminotransferase (ALT), aspartate Aminotransferase (AST), glucose, urea, creatinine, potassium, and phosphate and significant decreases (P≤0.05) in albumin, cholesterol, sodium, chloride, and calcium levels, where serum level of magnesium did not show any significant difference between the groups. In addition, oxidative stress was present as there was a significant decrease in reduced glutathione (GSH) level and superoxide dismutase (SOD) activity, and a significant increase in malondialdehyde (MDA) level.

**Background:**

These findings indicate a pathogenesis cascade whereby ingestion of poorly digested food causes ruminal stasis, systemic acidosis, liver dysfunction, renal insufficiency, electrolyte disturbances, oxidative stress and oxidative stress. Using a single biomarker may underestimate the true severity of the disease, while combining biochemical, electrolyte and oxidative measurements provides a more complete picture.

## Introduction

Ruminal impaction is a severe digestive condition in ruminants caused by excessive accumulation and hardening of ingesta in the rumen, leading to distention and functional stasis. In sheep, this is generally related to the ingestion of poor-quality, fibrous, or indigestible feed, leading to short-chain fatty acid (SCFA) generation, which can exceed absorption, and ruminal pH becomes depressed, especially in periods of nutritional stress or inadequate management ( Elmeligy et al., 2025).

In addition to its mechanical effects, ruminal impaction induces a cascade of systemic disorders, including diarrhea, severe dehydration, electrolyte disturbances, acid-base imbalance, and endotoxemia, which all compromise animal health and welfare and contribute to significant economic losses in small ruminant production systems ( Constable et al., 2016; Voulgarakis et al., 2023).

The pathophysiological effects begin with interrupting normal ruminal motility and fermentation of the microflora. Alterations in the ruminal contents disrupt the normal symbiotic relationship between the host and its resident microflora, leading to an imbalance within the microbial community. This dysbiosis promotes the overgrowth of pathogenic species, releasing endotoxins into the digestive environment. At the same time, reduced food intake and fluid retention in the gastrointestinal tract lead to severe dehydration and haemoconcentration due to diarrhea ( Smith, 2014; Kumar et al., 2019).

The resulting hypovolaemia due to osmotic pressure impaired peripheral circulation and renal perfusion, predisposing the animal to pre-existing azotemia and altering electrolyte homeostasis, e.g., decreased concentration of strong cations (Lactic acid) or increased concentration of weak cations ( Molitoris, 2022). The central cause of the clinical defect in animals is biochemical and electrolyte disturbances ( Gał ę ska et al., 2022). Reduced renal filtration rate increases urea nitrogen (BUN) and creatinine blood levels, which reflect impaired renal function. Furthermore, decreased salivary secretion, usually the primary source of sodium bicarbonate and phosphate, contributes to systemic acidosis and electrolyte disturbances, particularly hyponatraemia, hyperkalaemia and hypochloremia ( Elnady et al., 2019). These ionic imbalances impair neuromuscular function and exacerbate systemic dysfunction, increasing the clinical impact due to an increase in the anion gap due to lactate acting as a non-measurable anion and lowering the measurable anion ( Hernández et al., 2014).

Oxidative stress is increasingly recognized as a key factor in ruminant gastrointestinal pathogenesis. Systemic inflammatory reactions and endotoxin release are associated with ruminal stasis, in particular lipopolysaccharides, which are released from the pathogenic species, stimulating the immune system and increasing the production of reactive oxygen species (ROS) ( Ayemele *et al*., 2021). When antioxidant protection is impaired, an imbalance leads to oxidative stress, lipoprotein peroxidation, and further cell damage ( Celi, 2011; Khamees *et al*., 2024).

Increased lipid peroxidation, represented by malondialdehyde (MDA), has been observed in acute rumen acidosis and associated digestive disorders, highlighting the role of oxidative damage in disease progression ( Zeng *et al*., 2023).

Although the recognition of ruminal impaction is increasing, comprehensive assessments of serum biochemical, electrolyte, and oxidative stress profiles in sheep with naturally occurring ruminal impaction are still uncommon. Systematic characterization of these parameters is necessary to improve diagnostic accuracy and guide treatment of fluid overload, electrolyte replacement, and antioxidant therapy. This is one of the first studies to integrate haematobiochemical, electrolyte, and oxidative markers into evaluating ruminal impaction in sheep. This approach should improve our understanding of its pathophysiology and help to develop more effective treatments.

## Materials and methods

A case-control study was conducted in 20 adult female sheep (Awassi Sheep Breeding) (10 in the impaction group and 10 in the control group); the animals in the impaction group were diagnosed in Fallujah, Iraq, based on clinical signs of anorexia, dehydration, diarrhoea, foamy ruminal contents, and pale mucous membranes ( Constable et al., 2016), confirmed by laboratory findings of hyperkalaemia. The control group comprised age, sex, and weight-matched clinically normal sheep.

The pooled venous blood was collected into tubes containing lithium heparin, centrifuged at 3000 x g for 15 min, and the plasma was pooled and frozen at -20°C for analysis. blood sampling, were performed in accordance with American Veterinary Medical Association (AVMA) guidelines ( Underwood and Raymond, 2013). Plasma biochemical parameters (albumin, cholesterol, urea, creatinine, alanine aminotransferase (ALT), and aspartate aminotransferase (AST) were measured using commercial colorimetric kits (Agape Diagnostics, Switzerland; Sam Diagnostics, United Arab Emirates; Biolab SAS, France). Electrolyte concentrations (sodium, potassium, chloride, calcium, magnesium, phosphate) were determined using an automatic chemical analyzer (Smart 150, Sam Diagnostic, UAE) and the corresponding commercial reagents. Oxidative stress biomarkers (superoxide dismutase (SOD), malondialdehyde (MDA), and reduced glutathione (GSH)) were quantified using ELISA kits (Biolab SAS, France). The data were checked for normality and analyzed by an independent t-test using SPSS (version 21.0; IBM). Data are expressed as mean ± SE, and statistical significance was set at P < 0.05 ( Larsen et al., 1973).

## Results

1. **Effect of metabolic acidosis on blood biochemical parameters in sheep.**
Table 1, showed that most serum biochemical parameters were significantly higher in the impaction group (P ≤ 0.05), while albumin and cholesterol were significantly lower (P ≤ 0.05).
2. **Effect of metabolic acidosis on blood electrolytes.**
  The result showed potassium and phosphate levels in serum were significantly increased ( Table 2) due to metabolic acidosis (Impaction), while sodium, calcium, and chloride levels were significantly decreased (p ≤ 0.05). Magnesium concentrations, on the other hand, did not change (P ≤ 0.05).
3. **Effect of metabolic acidosis on oxidative markers in the blood serum of sheep.**
  The impaction group’s ( Table 3) plasma antioxidant markers showed significant declines, with GSH and SOD activity significantly lower than controls (P ≤ 0.05). In contrast, there was a significant increase in MDA levels in the afflicted sheep (P ≤ 0.05).

**Table 1.** The effect of ruminal impaction (metabolic acidosis) on blood biochemical (alanine aminotransferase (ALT), aspartate Aminotransferase (AST), glucose, cholesterol, albumin, urea, and creatinine) values in local Iraqi sheep.

Parameters	Control group	Impaction group	LSD
ALT (IU/l)	27 ± 1.7 B	34.6 ± 1.4 A	4.63
AST (IU/l)	30.6 ± 1.5 B	37.5 ± 1.2 A	4.03
Glucose (mg/dl)	72.6 ± 1.9 B	100.8 ± 2.1A	5.94
Cholesterol (mg/dl)	86.4 ± 1.7 A	64.8 ± 2 B	5.51
Albumin (g/dl)	5 ± 0.18A	3.34 ± 0.3 B	0.74
Urea (mg/dl)	26.6 ± 1.3 B	44.4 ± 2.2 A	5.4
Creatinine (mg/dl)	1.08 ± 0.09 B	2.2 ± 0.07 A	0.24

-Number of animals in each group (10), Data reflected as Mean ± Standard Error (SE).

-The different capital letters indicate significant changes (P ≤ 0.05) between groups variation.

**Table 2.** Effect of ruminal impaction (metabolic acidosis) blood electrolyte (sodium, potassium chloride, calcium, phosphate, and magnesium) values in local Iraqi sheep.

Parameters	Control group	Impaction group	LSD
Na (mmol/L)	143.4 ± 3.2 A	122.6 ± 2.9 B	9.14
K (mmol/L)	4.42 ± 0.19 B	6.94 ± 0.46A	1.04
Cl (mmol/L)	102 ± 2.9 A	90.4 ± 2.7 B	8.32
Ca (mg/dl)	20 ± 0.9 A	16.7 ± 1.2 B	3.24
Phosphate (U/L)	2.5 ± 0.14 B	10.5 ± 0.6 A	1.3
Mg (mg/dl)	2.23 ± 0.09 A	2.22 ± 0.1 A	0.28

-Number of animals in each group (10), Data reflected as Mean ± Standard Error (SE).

-The different capital letters indicate significant (P ≤ 0.05) changes between groups variation.

**Table 3.** The ruminal impaction (metabolic acidosis) effects on oxidative markers (reduced glutathione (GSH), superoxide dismutase (SOD), and malondialdehyde (MDA)) in the blood of local Iraqi sheep.

Parameters	Control group	Impaction group	LSD
GSH (μmol/l)	9.2 ± 1.1 A	4.2 ± 1 B	3.12
SOD (U/ml)	19. 2± 1.3 **A**	10.8 ± 1.1 **B**	3.58
MDA (μmol/l)	9.11 ± 0.91 **B**	14.15 ± 0.9 **A**	2.9

-Number of animals in each group (10), Data reflected as Mean ± Standard Error (SE).

-The different capital letters indicate significant (P ≤ 0.05) changes between groups variation.

## Discussion

This study shows a coordinated pattern of biochemical, electrolyte, and oxidative markers in metabolic acidosis secondary to ruminal impaction in sheep. Elevated serum ALT and AST levels ( Table 1) indicate liver damage, most likely caused by lactic acid and other toxic fermentation products, and cause liver abscesses, pulmonary or renal damage ( Manosalva et al., 2022), and are consistent with previous reports of elevated liver enzymes and decreased hepatic protein synthesis in sheep with acute ruminant acidosis ( Alkabi *et al*., 2019; Elmeligy *et al*., 2025). In addition, the hyperglycemia observed in the impaction group resulted in elevated lactic acid, which seems to reflect a systemic stress response, as metabolic acidosis stimulates the release of catecholamine and cortisol, stimulating gluconeogenesis ( Zhang *et al*., 2019). Similar glycaemic events have been documented in ruminants under dietary and acidotic stress ( Wang *et al*., 2025).

In addition to these changes, decreased serum albumin and cholesterol indicate impaired hepatic synthesis. On the other hand, hypoalbuminemia reduces plasma osmotic pressure and buffer capacity. In contrast, hypocholesterolaemia indicates impaired lipid metabolism under excessive stress on the liver due to the accumulation of D. lactate, supporting the apparent findings of elevated liver enzymes. These observations are consistent with previous findings of decreased total protein and albumin in ruminal acidosis and highlight the magnitude of liver dysfunction ( Zhang et al., 2023). Furthermore, increased lactate production impairs the kidney’s ability to convert lactate into glucose ( DeFronzo et al., 2012), adding to elevated urea and creatinine levels, which indicate renal involvement, most likely due to a decrease in glomerular filtration rate and reduced renal perfusion, both of which are exacerbated by dehydration and metabolic acidosis. Comparable elevations in these parameters have been reported in sheep affected by ruminal acidosis, reinforcing the view that renal dysfunction is a frequent complication of the condition ( Zheng et al., 2024).

Electrolyte disturbances characterized by metabolic acidosis ( Matyukhin et al., 2020) have been clearly observed, with increases in serum potassium and phosphate levels, and decreases in sodium and calcium ( Table 2). These ionic shifts are attributed mainly to intracellular buffering of excess hydrogen ions, which causes the efflux of potassium to the extracellular space. When acidosis develops in cells, Na ^+^-K ^+^pump (ATPase pump) activity is reduced and the pH and phosphorus levels are altered, interfering with calcium binding and intestinal absorption. The resulting hypocalcemia may also reflect D-lactate accumulation in the intestinal mucosa, where a mucosal injury may further reduce calcium absorption. These results are consistent with this explanation; Kumar et al. (2019) reported a comparable decrease in serum calcium levels in ruminant cows with rumen acidosis. These changes have important physiological consequences, particularly for cardiac function and neuromuscular excitability.

Oxidative stress is a significant factor in the pathophysiology of ruminant acidosis. The reduction in glutathione (GSH) and superoxide dismutase (SOD) and the increase in malondialdehyde (MDA) ( Table 3) indicate a weakened antioxidant protection. These amendments align with the Kirbaş et al. (2014), which reported that acute ruminal acidosis promotes the production of excess reactive oxygen species (ROS), depleting the antioxidant capacity of the cells and speeding up lipid peroxidation. Excessive ROS production may be due to microbial dysbiosis, impaired ruminal fermentation, and systemic inflammatory reactions, exacerbating oxidative damage ( Lian et al., 2024). Clinically, oxidative stress may impair organ function and neuromuscular performance, which underlines the importance of monitoring antioxidant markers in addition to biochemical and electrolyte parameters.

## Conclusion

These findings suggest a pathophysiological cascade in which ingestion of poorly digested food causes ruminal stasis, systemic acidosis, hepatic dysfunction, renal impairment, electrolyte disturbances, oxidative stress, and electrolyte disturbances. Relying on a single biomarker may underestimate the actual severity of the disease, while a combination of biochemical, electrolyte, and oxidative measurements provides a more comprehensive picture. In clinical practice, early detection using multiple markers is crucial, and early correction of potassium and calcium imbalances may be paramount for preventing complications. In addition, supportive treatment targeting liver, renal, and oxidative function is required to reduce systemic damage and to improve overall results.

## Ethical considerations

All experimental procedures were conducted per the guidelines approved by the Scientific Research Ethics Committee of the Faculty of Veterinary Medicine, University of Fallujah (Approval No. 4; 16 September 2025).

## Data Availability

Underlying data Zenodo: Dataset for the ruminal impaction in sheep (2025). https://doi.org/10.5281/zenodo.18075931.( Abed *et al*., 2025). **Arrive checklist**: https://doi.org/10.5281/zenodo.18075931 ( Abed *et al*., 2025). Data are available under the terms of the Creative Commons Zero “No rights reserved” data waiver (CC0 1.0 Public domain dedication).
