# Physiological Alterations in Local Iraqi Sheep Affected by Ruminal Impaction: Insights from Biochemical, Electrolyte, and Oxidative Markers

**DOI:** 10.12688/f1000research.174402.2

**Published:** 2026-03-31

**Authors:** Sabea Khamees Abed, Mustafa A. Saud, Ahmed Emad Abood, Maysam Abdulrahman Ghazi

**Affiliations:** 1 College of Veterinary Medicine, University of Fallujah, Al-Fallujah, Al Anbar Governorate, Iraq

**Keywords:** Keywords: ruminal impaction; sheep; metabolic acidosis; oxidative stress

## Abstract

**Background:**

Ruminal impaction is a serious digestive disorder in sheep, leading to systemic metabolic acidosis and, as a consequence, widespread physiological disturbances. Thus, the objective of this study was to estimate biochemical, electrolyte and oxidative parameters in a local Iraqi sheep that was suffering from ruminal impaction.

**Methods:**

A case control study was performed in 20 adult female sheep (Awassi sheep breeder) (10 ruminal impaction and 10 control) diagnosed with in Fallujah, Iraq, based on clinical signs and confirmed by laboratory findings of hyperkalaemia. The control group consisted of sheep of age, sex and weight consistent with clinically normal sheep. At the end of the experiment, blood samples were collected, and serum was analyzed for electrolytes, biochemical and oxidative markers using established laboratory methods.

**Results:**

The impacted animals showed significant increases (P≤0.05) in alanine aminotransferase (ALT), aspartate Aminotransferase (AST), glucose, urea, creatinine, potassium, and phosphate and significant decreases (P≤0.05) in albumin, cholesterol, sodium, chloride, and calcium levels, where serum level of magnesium did not show any significant difference between the groups. In addition, oxidative stress was present as there was a significant decrease in reduced glutathione (GSH) level and superoxide dismutase (SOD) activity, and a significant increase in malondialdehyde (MDA) level.

**Background:**

These findings indicate a pathogenesis cascade whereby ingestion of poorly digested food causes ruminal stasis, systemic acidosis, liver dysfunction, renal insufficiency, electrolyte disturbances, oxidative stress and oxidative stress. Using a single biomarker may underestimate the true severity of the disease, while combining biochemical, electrolyte and oxidative measurements provides a more complete picture.

## Introduction

Ruminal impaction is a severe digestive condition in ruminants caused by excessive accumulation and hardening of ingesta in the rumen, leading to distention and functional stasis. In sheep, this is generally related to the ingestion of poor-quality, fibrous, or indigestible feed, leading to short-chain fatty acid (SCFA) generation, which can exceed absorption, and ruminal pH becomes depressed, especially in periods of nutritional stress or inadequate management ( Elmeligy *et al.*, 2025).

In addition to its mechanical effects, ruminal impaction induces a cascade of systemic disorders, including diarrhea, severe dehydration, electrolyte disturbances, acid-base imbalance, and endotoxemia, which all compromise animal health and welfare and contribute to significant economic losses in small ruminant production systems ( Constable *et al.*, 2016; Voulgarakis *et al.*, 2023).

The pathophysiological effects begin with interrupting normal ruminal motility and fermentation of the microflora. Alterations in the ruminal contents disrupt the normal symbiotic relationship between the host and its resident microflora, leading to an imbalance within the microbial community. This dysbiosis promotes the overgrowth of pathogenic species, releasing endotoxins into the digestive environment. At the same time, reduced food intake and fluid retention in the gastrointestinal tract lead to severe dehydration and haemoconcentration due to diarrhea ( Smith, 2014; Kumar *et al.*, 2019).

The resulting hypovolaemia due to osmotic pressure impaired peripheral circulation and renal perfusion, predisposing the animal to pre-existing azotemia and altering electrolyte homeostasis, e.g., decreased concentration of strong cations (Lactic acid) or increased concentration of weak cations ( Molitoris, 2022). The central cause of the clinical defect in animals is biochemical and electrolyte disturbances ( Gał ę ska *et al.*, 2022). Reduced renal filtration rate increases urea nitrogen (BUN) and creatinine blood levels, which reflect impaired renal function. Furthermore, decreased salivary secretion, usually the primary source of sodium bicarbonate and phosphate, contributes to systemic acidosis and electrolyte disturbances, particularly hyponatraemia, hyperkalaemia and hypochloremia ( Elnady *et al.*, 2019). These ionic imbalances impair neuromuscular function and exacerbate systemic dysfunction, increasing the clinical impact due to an increase in the anion gap due to lactate acting as a non-measurable anion and lowering the measurable anion ( Hernández *et al.*, 2014).

Oxidative stress is increasingly recognized as a key factor in ruminant gastrointestinal pathogenesis. Systemic inflammatory reactions and endotoxin release are associated with ruminal stasis, in particular lipopolysaccharides, which are released from the pathogenic species, stimulating the immune system and increasing the production of reactive oxygen species (ROS) ( Ayemele *et al*., 2021). When antioxidant protection is impaired, an imbalance leads to oxidative stress, lipoprotein peroxidation, and further cell damage ( Celi, 2011; Khamees *et al*., 2024).

Increased lipid peroxidation, represented by malondialdehyde (MDA), has been observed in acute rumen acidosis and associated digestive disorders, highlighting the role of oxidative damage in disease progression ( Zeng *et al*., 2023).

Although the recognition of ruminal impaction is increasing, comprehensive assessments of serum biochemical, electrolyte, and oxidative stress profiles in sheep with naturally occurring ruminal impaction are still uncommon. Systematic characterization of these parameters is necessary to improve diagnostic accuracy and guide treatment of fluid overload, electrolyte replacement, and antioxidant therapy. This is one of the first studies to integrate haematobiochemical, electrolyte, and oxidative markers into evaluating ruminal impaction in sheep. This approach should improve our understanding of its pathophysiology and help to develop more effective treatments.

## Materials and methods

The study involved clinically examined Awassi sheep aged approximately 2-3 years. All animals were non-pregnant and non-lactating females and were maintained under identical care and feeding conditions (10 in the impaction group and 10 in the control group). This group included animals with natural clinical cases of ruminal impaction, admitted to a veterinary clinic and diagnosed based on clinical examination and rumen condition data in Fallujah, Iraq, based on clinical signs of anorexia, dehydration, diarrhoea, foamy ruminal contents, and pale mucous membranes ( Constable *et al.*, 2016). The control group comprised age, sex, and weight-matched clinically normal animals whose biochemical values were within the physiological reference range reported for sheep. The venous blood samples were taken from each animal using sterile tubing containing lithium heparin, centrifuged at 3000 x g for 15 min, and the plasma was pooled and frozen at -20°C for analysis. Blood sampling, were performed in accordance with American Veterinary Medical Association (AVMA) guidelines ( Underwood and Raymond, 2013). Plasma biochemical parameters (albumin, cholesterol, urea, creatinine, alanine aminotransferase (ALT), and aspartate aminotransferase (AST) were measured using commercial colorimetric kits (Agape Diagnostics, Switzerland; Sam Diagnostics, United Arab Emirates; Biolab SAS, France). Electrolyte concentrations (sodium, potassium, chloride, calcium, magnesium, phosphate) were determined using an automatic chemical analyzer (Smart 150, Sam Diagnostic, UAE) and the corresponding commercial reagents. Oxidative stress biomarkers (superoxide dismutase (SOD), malondialdehyde (MDA), and reduced glutathione (GSH)) were quantified using ELISA kits (Biolab SAS, France). Statistical analysis was carried out with SPSS (version 21.0; IBM). Data are presented as mean ± standard error. Significance was assessed using the Shapiro-Wilk test. Differences between the occlusion and control groups were analyzed using an independent two-sample t-test. To reduce the risk of type I errors associated with multiple comparisons, the false-positive rate (FDR) was tested using the Benjamini-Hochberg test, and the significance level was set at P ≤ 0.05 ( Larsen *et al.*, 1973).

## Results

1. **Effect of ruminal impaction on blood biochemical parameters in sheep.**
Table 1, showed that most serum biochemical parameters were significantly higher in the impaction group (P ≤ 0.05), while albumin and cholesterol were significantly lower (P ≤ 0.05).
2. **Effect of ruminal impaction on blood electrolytes.**
  The result showed potassium and phosphate levels in serum were significantly increased ( Table 2) due to metabolic acidosis (Impaction), while sodium, calcium, and chloride levels were significantly decreased (P ≤ 0.05). Magnesium concentrations, on the other hand, did not change (P ≤ 0.05).
3. **Effect of ruminal impaction on oxidative markers in the blood serum of sheep.**
  The impaction group’s ( Table 3) plasma antioxidant markers showed significant declines, with GSH and SOD activity significantly lower than controls (P ≤ 0.05). In contrast, there was a significant increase in MDA levels in the afflicted sheep (P ≤ 0.05).

**Table 1.** The effect of ruminal impaction on blood biochemical (alanine aminotransferase (ALT), aspartate Aminotransferase (AST), glucose, cholesterol, albumin, urea, and creatinine) values in local Iraqi sheep.

Parameters	Control group	Impaction group	P-value
ALT (IU/l)	27 ± 1.7 B	34.6 ± 1.4 A	0.00037
AST (IU/l)	30.6 ± 1.5 B	37.5 ± 1.2 A	0.00015
Glucose (mg/dl)	72.6 ± 1.9 B	100.8 ± 2.1A	8.5*10 ^−12^
Cholesterol (mg/dl)	86.4 ± 1.7 A	64.8 ± 2 B	2.19*10 ^−12^
Albumin (g/dl)	5 ± 0.18A	3.34 ± 0.3 B	1.52*10 ^−9^
Urea (mg/dl)	26.6 ± 1.3 B	44.4 ± 2.2 A	5.27*10 ^−10^
Creatinine (mg/dl)	1.08 ± 0.09 B	2.2 ± 0.07 A	5.71*10 ^−17^

-Number of animals in each group (10), Data reflected as Mean ± Standard Error (SE).

-The different capital letters indicate significant changes (P ≤ 0.05) between groups variation.

**Table 2.** Effect of ruminal impaction blood electrolyte (sodium, potassium chloride, calcium, phosphate, and magnesium) values in local Iraqi sheep.

Parameters	Control group	Impaction group	P-value
Na (mmol/L)	143.4 ± 3.2 A	122.6 ± 2.9 B	1.4*10 ^−7^
K (mmol/L)	4.42 ± 0.19 B	6.94 ± 0.46 A	1.62*10 ^−8^
Cl (mmol/L)	102 ± 2.9 A	90.4 ± 2.7 B	0.0001
Ca (mg/dl)	20 ± 0.9 A	16.7 ± 1.2 B	0.0002
Phosphate (mmol/L)	2.5 ± 0.14 B	10.5 ± 0.6 A	8.7*10 ^−16^
Mg (mg/dl)	2.23 ± 0.09 A	2.22 ± 0.1 A	0.01

-Number of animals in each group (10), Data reflected as Mean ± Standard Error (SE).

-The different capital letters indicate significant (P ≤ 0.05) changes between groups variation.

**Table 3.** The ruminal impaction effects on oxidative markers (reduced glutathione (GSH), superoxide dismutase (SOD), and malondialdehyde (MDA)) in the blood of local Iraqi sheep.

Parameters	Control group	Impaction group	P-value
GSH (μmol/l)	9.2 ± 1.1 A	4.2 ± 1 B	0.056
SOD (U/ml)	19. 2± 1.3 **A**	10.8 ± 1.1 **B**	4.56*10 ^−5^
MDA (μmol/l)	9.11 ± 0.91 **B**	14.15 ± 0.9 **A**	0.003

-Number of animals in each group (10), Data reflected as Mean ± Standard Error (SE).

-The different capital letters indicate significant (P ≤ 0.05) changes between groups variation.

## Discussion

Although metabolic acidosis has been reported in ruminal impaction, direct acid-base parameters such as blood pH, bicarbonate concentration or blood gas analysis have not been measured in this study. Therefore, the current findings should be interpreted as biochemical changes possibly related to acid-base imbalance and not as a confirmed diagnosis of metabolic acidosis.

So that, this study shows a coordinated pattern of biochemical, electrolyte, and oxidative markers in metabolic acidosis secondary to ruminal impaction in sheep. Elevated serum ALT and AST levels ( Table 1) indicate liver damage, most likely may indicated by lactic acid and other toxic fermentation products, and may indicate liver abscesses, pulmonary or renal damage ( Manosalva *et al.*, 2022), and are consistent with previous reports of elevated liver enzymes and decreased hepatic protein synthesis in sheep with acute ruminant acidosis ( Alkabi *et al*., 2019; Elmeligy *et al*., 2025). In addition, the hyperglycemia observed in the impaction group may contribute to elevate lactic acid, which seems to reflect a systemic stress response, as metabolic disturbance related with acidosis stimulates the release of catecholamine and cortisol, stimulating gluconeogenesis ( Zhang *et al*., 2019). Similar glycaemic events have been documented in ruminants under dietary and acidotic stress ( Wang *et al*., 2025).

In addition to these changes, decreased serum albumin and cholesterol indicate impaired hepatic synthesis. On the other hand, hypoalbuminemia reduces plasma osmotic pressure and buffer capacity. In contrast, hypocholesterolaemia indicates impaired lipid metabolism under excessive stress on the liver may be due to the accumulation of D. lactate, supporting the apparent findings of elevated liver enzymes. These observations are consistent with previous findings of decreased total protein and albumin in ruminal acidosis and highlight the magnitude of liver dysfunction ( Zhang *et al.*, 2023). Furthermore, increased lactate production impairs the kidney’s ability to convert lactate into glucose ( DeFronzo *et al.*, 2012), adding to elevated urea and creatinine levels, which indicate renal involvement, most likely may be due to a decrease in glomerular filtration rate and reduced renal perfusion, both of which are exacerbated by dehydration and metabolic disturbance associated with acidosis. Comparable elevations in these parameters have been reported in sheep affected by ruminal acidosis, reinforcing the view that renal dysfunction is a frequent complication of the condition ( Zheng *et al.*, 2024).

Electrolyte disturbances characterized by metabolic disturbance associated with acidosis ( Matyukhin *et al.*, 2020) have been clearly observed, with increases in serum potassium and phosphate levels, and decreases in sodium and calcium ( Table 2). These ionic shifts are attributed mainly to intracellular buffering of excess hydrogen ions, which causes the efflux of potassium to the extracellular space. When acidosis develops in cells, Na ^+^-K ^+^pump (ATPase pump) activity is consistent with reduced and the pH and phosphorus levels are altered, interfering with calcium binding and intestinal absorption. The resulting hypocalcemia may be associated with D-lactate accumulation in the intestinal mucosa, where a mucosal injury may further reduce calcium absorption. These results are consistent with this explanation; Kumar *et al.* (2019) reported a comparable decrease in serum calcium levels in ruminant cows with rumen acidosis. These changes have important physiological consequences, particularly for cardiac function and neuromuscular excitability.

Oxidative stress is a significant factor in the pathophysiology of ruminant acidosis. The reduction in glutathione (GSH) and superoxide dismutase (SOD) and the increase in malondialdehyde (MDA) ( Table 3) indicate a weakened antioxidant protection. These amendments align with the Kirbaş *et al.* (2014), which reported that acute ruminal acidosis promotes the production of excess reactive oxygen species (ROS), depleting the antioxidant capacity of the cells and speeding up lipid peroxidation. Excessive ROS production may be due to microbial dysbiosis, impaired ruminal fermentation, and systemic inflammatory reactions, exacerbating oxidative damage ( Lian *et al.*, 2024). Clinically, oxidative stress may impair organ function and neuromuscular performance, which underlines the importance of monitoring antioxidant markers in addition to biochemical and electrolyte parameters.

## Conclusion

These findings may support a multifactorial metabolic disturbance in which ingestion of poorly digested food causes ruminal stasis, systemic acidosis, hepatic dysfunction, renal impairment, electrolyte disturbances, oxidative stress, and electrolyte disturbances. Relying on a single biomarker may underestimate the actual severity of the disease, while a combination of biochemical, electrolyte, and oxidative measurements provides a more comprehensive picture. In clinical practice, early detection using multiple markers is crucial, and early correction of potassium and calcium imbalances may be paramount for preventing complications. In addition, supportive treatment targeting liver, renal, and oxidative function is required to reduce systemic damage and to improve overall results.

## Ethical considerations

All experimental procedures were conducted per the guidelines approved by the Scientific Research Ethics Committee of the Faculty of Veterinary Medicine, University of Fallujah (Approval No. 4; 16 September 2025).

## Data Availability

Underlying data Zenodo: Dataset for the ruminal impaction in sheep (2025). https://doi.org/10.5281/zenodo.18075931.( Abed *et al*., 2025). **Arrive checklist**: https://doi.org/10.5281/zenodo.18075931 ( Abed *et al*., 2025). Data are available under the terms of the Creative Commons Zero “No rights reserved” data waiver (CC0 1.0 Public domain dedication).
