# Supplementary material for: Physiological Alterations in Local Iraqi Sheep Affected by Ruminal Impaction: Insights from Biochemical, Electrolyte, and Oxidative Markers
Source: F1000Res. 2026 Mar 31;15:110. Originally published 2026 Jan 23. [Version 2] doi: 10.12688/f1000research.174402.2 (PMC13100609; doi:10.12688/f1000research.174402.2)
Supplement: Peer review history

# Reviewer report: Reviewer response for version 2

**DOI:** 10.5256/f1000research.197497.r472403

**Published:** 2026-04-21

**Authors:** Masar Jabbar Al-kurdy

**Affiliations:** 1 AL-Furat AL- Awsat Technical University, Najaf, Iraq

Multiple testing: 

 The study compares 13 parameters (ALT, AST, glucose, cholesterol, albumin, urea, creatinine, Na, K, Cl, Ca, phosphate, Mg, plus 3 oxidative markers – effectively 16 tests) using independent t‑tests at α = 0.05.

 the family‑wise error rate is ~56% (1 − 0.95^16). Many significant findings could be false positives.

 -Reporting of LSD: Tables 1–3 present “LSD” (least significant difference) values, which are not standard for independent t‑tests. This suggests a potential confusion with post‑hoc tests from ANOVA. The statistical section should describe exactly which test was used (t‑test or ANOVA) and report actual p‑values or adjusted thresholds, not LSD.

 -Normality testing: Only stated as “checked for normality” without naming the test (Shapiro‑Wilk?) or reporting results.

 Phosphate Units – Erroneous (Major)

 * Table 2 reports Phosphate (U/L). Phosphate concentration in serum is measured in mmol/L or mg/dL – not in enzymatic units (U/L). The control value (2.5 U/L) and impaction value (10.5 U/L) are biologically implausible for phosphate. This appears to be a typographical or transcription error.

 Required action: Verify the correct units (likely mg/dL or mmol/L) and correct the table. Also verify the numerical values – a four‑fold increase (2.5 to 10.5) is extreme but possible; however, the units must be correct.

Is the work clearly and accurately presented and does it cite the current literature?

Yes

If applicable, is the statistical analysis and its interpretation appropriate?

Partly

Are all the source data underlying the results available to ensure full reproducibility?

No source data required

Is the study design appropriate and is the work technically sound?

Yes

Are the conclusions drawn adequately supported by the results?

Yes

Are sufficient details of methods and analysis provided to allow replication by others?

Yes

Reviewer Expertise:

endocrine disorder, nanoparticles,

I confirm that I have read this submission and believe that I have an appropriate level of expertise to confirm that it is of an acceptable scientific standard.

# Reviewer report: Reviewer response for version 1

**DOI:** 10.5256/f1000research.192297.r460234

**Published:** 2026-02-28

**Authors:** Jinxin Liu

**Affiliations:** 1 Laboratory of Gastrointestinal Microbiology, Jiangsu Key Laboratory of Gastrointestinal Nutrition and Animal Health, College of Animal Science and Technology, Nanjing Agricultural University, Nanjing, China

This manuscript investigates biochemical, electrolyte, and oxidative stress alterations in local Iraqi Awassi sheep affected by ruminal impaction using a case–control design. The study addresses a clinically relevant condition in small ruminant production and attempts to integrate metabolic, hepatic, renal, electrolyte, and oxidative parameters into a unified pathophysiological framework. The inclusion of oxidative stress markers alongside routine biochemical indices adds value and broadens the diagnostic perspective.

 However, while the study is clinically meaningful, several methodological and interpretational aspects require clarification. The sample characterization lacks sufficient detail, statistical methodology is overly simplified given the number of comparisons performed, and some mechanistic explanations in the discussion appear speculative relative to the data presented. Addressing these issues would improve scientific rigor and reproducibility while strengthening the translational value of the findings.

 Major Comments

 1. Study design and case definition require clearer characterization

 Although the study is described as a case–control design (page 3), several key elements remain unclear:

- The age range, reproductive status (parity), and physiological stage of the sheep are not specified. These factors can significantly influence biochemical and oxidative parameters.
- It is not clearly stated whether ruminal impaction was naturally occurring or experimentally induced. The manuscript implies natural cases, but this should be explicitly clarified.
- Hyperkalaemia is described as confirmatory for diagnosis; however, potassium elevation is also one of the outcome variables. This raises concerns regarding circular reasoning in case definition.

The authors should provide a more detailed clinical characterization of cases and clearly define diagnostic criteria independent of the primary outcome measures.

 2. Statistical approach and multiple comparisons

 The study compares numerous biochemical, electrolyte, and oxidative markers using independent t-tests with a significance threshold of P < 0.05. Given the number of parameters assessed (ALT, AST, glucose, cholesterol, albumin, urea, creatinine, six electrolytes, and three oxidative markers), the risk of type I error is substantial.

 The authors should clarify:

- Whether assumptions of normality and variance homogeneity were formally tested.
- Whether any correction for multiple comparisons was considered.
- Why LSD values are reported in tables despite using independent t-tests.

A more robust statistical framework or at least justification of the current approach would enhance credibility.

 3. Interpretation of causality and mechanistic claims

 The discussion proposes a pathophysiological cascade linking ruminal impaction to systemic acidosis, hepatic dysfunction, renal impairment, electrolyte imbalance, and oxidative stress (pages 5–6). While the observed associations are plausible, the study design does not allow causal inference.

 Several mechanistic explanations (e.g., D-lactate–mediated hepatic injury, Na⁺/K⁺-ATPase suppression, lactate-driven renal gluconeogenesis impairment) are presented as explanatory conclusions but were not directly measured in this study.

 The discussion should be reframed to clearly distinguish between observed associations and hypothesized mechanisms based on prior literature.

 Minor Comments

1. Pooling of blood samples (page 3)
  The methods state that pooled venous blood was collected and plasma was pooled before freezing. It is unclear whether pooling occurred within individuals or across individuals. If samples were pooled across animals, this would invalidate statistical comparisons. This must be clarified.
2. Reference to metabolic acidosis
  The manuscript repeatedly refers to “metabolic acidosis,” yet no direct measurements of blood pH, bicarbonate, base excess, or blood gas analysis are provided. The authors should clarify how metabolic acidosis was confirmed.
3. Phosphate units (Table 2)
  Phosphate is reported in U/L, which appears inconsistent with standard electrolyte reporting (typically mmol/L or mg/dL). Units should be verified.

Is the work clearly and accurately presented and does it cite the current literature?

Yes

If applicable, is the statistical analysis and its interpretation appropriate?

Yes

Are all the source data underlying the results available to ensure full reproducibility?

No source data required

Is the study design appropriate and is the work technically sound?

Yes

Are the conclusions drawn adequately supported by the results?

Yes

Are sufficient details of methods and analysis provided to allow replication by others?

Yes

Reviewer Expertise:

ruminant nutrition/gut microbiota

I confirm that I have read this submission and believe that I have an appropriate level of expertise to confirm that it is of an acceptable scientific standard, however I have significant reservations, as outlined above.

# Response

**Published:** 2026-03-06

**Authors:** Sabea Abed

**Affiliations:** veterinary medicine, University of Fallujah, Al-Fallujah, Al Anbar Governorate, Iraq

**Dear Reviewer, Dr. Jinxin Liu**

 We sincerely thank you for your careful evaluation of our manuscript and for your constructive and insightful comments. Your suggestions were extremely valuable and have significantly improved the clarity, scientific rigor, and overall quality of the manuscript. All comments have been carefully considered, and the manuscript has been revised accordingly. Our detailed responses to each comment are provided below.

**Comment 1: Study design and case definition require clearer characterization**

 Thank you for this important observation. The manuscript has been revised to provide additional information regarding the clinical characterization of the animals, including age range and physiological status. We have also clarified that ruminal impaction cases were naturally occurring. Furthermore, the diagnostic criteria have been revised to ensure that potassium levels were not used as a confirmatory diagnostic parameter, thereby avoiding potential circular reasoning.

**Comment 2: Statistical approach and multiple comparisons**

 We appreciate this valuable suggestion. To strengthen the statistical rigor of the study, the statistical analysis section has been revised. Normality of the data was assessed using the Shapiro–Wilk test, and differences between groups were analyzed using an independent samples t-test. Additionally, to control for potential type I error due to multiple comparisons, the false discovery rate (FDR) was adjusted using the Benjamini–Hochberg procedure. The statistical analysis section has been updated accordingly, and the tables were revised to remove LSD values and report appropriate p-values.

**Comment 3: Interpretation of causality and mechanistic claims**

 We thank the reviewer for this insightful comment. The discussion has been carefully revised to avoid causal interpretations. The mechanistic explanations are now presented as hypotheses supported by previous literature rather than direct conclusions derived from the current data.

**Minor Comment 1: Pooling of blood samples**

 We apologize for the lack of clarity. The manuscript has been revised to clarify that blood samples were collected and processed individually for each animal. No pooling occurred across animals, and each sample was analyzed separately.

**Minor Comment 2: Reference to metabolic acidosis**

 We appreciate this comment. The discussion has been revised to clarify that metabolic acidosis was inferred based on previously reported pathophysiological mechanisms associated with ruminal disorders, rather than being directly measured in this study. This limitation has now been explicitly acknowledged in the revised manuscript.

**Minor Comment 3: Phosphate units**

 The units for phosphate have been carefully reviewed and corrected in the revised tables.

 Once again, we sincerely thank the reviewer for the valuable feedback, which has greatly improved the quality and clarity of our manuscript.

 Sincerely            ,

 Sabea Khamees Abed

# Reviewer report: Reviewer response for version 1

**DOI:** 10.5256/f1000research.192297.r453401

**Published:** 2026-02-09

**Authors:** Şevket Evci

**Affiliations:** 1 Kırıkkale University, Kırıkkale, Turkey

The materials section of the article lacks sufficient information regarding the sheep's age, number of births, and whether the impaction was experimentally induced. Furthermore, the normal range for blood values ​​is not specified, and comparisons are made using values ​​from a control group. Were the blood values ​​of the control group normal?

Is the work clearly and accurately presented and does it cite the current literature?

Yes

If applicable, is the statistical analysis and its interpretation appropriate?

Partly

Are all the source data underlying the results available to ensure full reproducibility?

Yes

Is the study design appropriate and is the work technically sound?

Yes

Are the conclusions drawn adequately supported by the results?

Yes

Are sufficient details of methods and analysis provided to allow replication by others?

Partly

Reviewer Expertise:

Nutritional disease of animals

I confirm that I have read this submission and believe that I have an appropriate level of expertise to confirm that it is of an acceptable scientific standard, however I have significant reservations, as outlined above.

# Response

**Published:** 2026-03-06

**Authors:** Sabea Abed

**Affiliations:** veterinary medicine, University of Fallujah, Al-Fallujah, Al Anbar Governorate, Iraq

**Dear Reviewer, Dr. Şevket Evci** **,**

 We sincerely appreciate the time and effort you devoted to evaluating our manuscript. Your constructive and insightful comments have been extremely valuable in improving the clarity, methodological rigor, and overall quality of the study. We have carefully considered all of your suggestions and revised the manuscript accordingly. Detailed responses to each of your comments are provided below.

 Additional information regarding the animals has been added to the Materials section, including age range and physiological status. The ruminal impaction cases were naturally occurring clinical cases and were not experimentally induced. Furthermore, the control group consisted of clinically healthy animals, and their biochemical values fell within the physiological reference ranges reported for sheep.

 Once again, we sincerely thank the reviewer for the valuable feedback, which has greatly improved the quality and clarity of our manuscript.

 Sincerely            ,

 Sabea Khamees Abed
